# Extracellular vesicles from IFN-γ-primed mesenchymal stem cells repress atopic dermatitis in mice

**DOI:** 10.1186/s12951-022-01728-8

**Published:** 2022-12-10

**Authors:** Jimin Kim, Seul Ki Lee, Minyoung Jung, Seon-Yeong Jeong, Haedeun You, Ji-Yeon Won, Sang-Deok Han, Hye Jin Cho, Somi Park, Joonghoon Park, Tae Min Kim, Soo Kim

**Affiliations:** 1 Brexogen Research Center, Brexogen Inc., Songpa-Gu, Seoul, 05855 South Korea; 2 Graduate School of International Agricultural Technology, Seoul National University, Pyeongchang, Gangwon-do 25354 South Korea; 3 Institutes of Green-Bio Science and Technology, Seoul National University, Pyeongchang, Gangwon-do 25354 South Korea

**Keywords:** Atopic dermatitis, iPSC-derived MSC, Extracellular vesicles, IFN-γ

## Abstract

**Background:**

Atopic dermatitis (AD) is a chronic inflammatory skin disorder characterized by immune dysregulation, pruritus, and abnormal epidermal barrier function. Compared with conventional mesenchymal stem cell (MSC), induced pluripotent stem cell (iPSC)-derived mesenchymal stem cell (iMSC) is recognized as a unique source for producing extracellular vesicles (EVs) because it can be obtained in a scalable manner with an enhanced homogeneity. Stimulation of iMSCs with inflammatory cytokines can improve the immune-regulatory, anti-inflammatory, and tissue-repairing potential of iMSC-derived EVs.

**Results:**

Proteome analysis showed that IFN-γ-iMSC-EVs are enriched with protein sets that are involved in regulating interferon responses and inflammatory pathways. In AD mice, expression of interleukin receptors for Th2 cytokines (IL-4Rα/13Rα1/31Rα) and activation of their corresponding intracellular signaling molecules was reduced. IFN-γ-iMSC-EVs decreased itching, which was supported by reduced inflammatory cell infiltration and mast cells in AD mouse skin; reduced IgE receptor expression and thymic stromal lymphopoietin and NF-kB activation; and recovered impaired skin barrier, as evidenced by upregulation of key genes of epidermal differentiation and lipid synthesis.

**Conclusions:**

IFN-γ-iMSC-EVs inhibit Th2-induced immune responses, suppress inflammation, and facilitate skin barrier restoration, contributing to AD improvement.

**Supplementary Information:**

The online version contains supplementary material available at 10.1186/s12951-022-01728-8.

## Background

Atopic dermatitis (AD) is a chronic, pruritic, uncontrolled inflammatory disease that affects 2–15% of the population worldwide, among which ~ 20% cases are moderate to severe [1]. The pathophysiology of AD has been attributed to epidermal barrier defects, immunological dysfunction, and nervous deregulation [2]. Among the various mediators, Th2-induced cytokines (e.g., IL-4, -13, and -31) are major contributing factors of AD; therefore, targeting Th2-mediated immune reactions has been one of the main strategies for AD therapy [3]. The current therapeutic options for AD include topical steroids, calcineurin inhibitors, anti-histamines, phosphodiesterase 4 inhibitors, JAK/STAT inhibitor, and systemic immunosuppression [4]. In addition, biologic therapeutics that antagonize the Th2 pathways as well as JAK activity have recently been approved for clinical use by the US Food and Drug Administration (FDA) for treating moderate to severe AD [5, 6]. Despite these recent advances, effective treatment for AD remains challenging due to the limited therapeutic responses and possible adverse effects [5, 7].

Mesenchymal stem cells (MSCs) are multipotent progenitor cells found in various connective tissues [8]. Due to their differentiation potential and immune-regulatory function, MSCs are now being recognized as a novel therapeutic tool for various immune or degenerative diseases [9–11]. However, several limitations need to be overcome to make MSCs clinically applicable. Most importantly, the growth competence of MSCs is gradually reduced, leading to replicative senescence [12]. Furthermore, engraftment of MSCs in vivo often fails mainly because they are often accumulated in the liver or lung and also because of the non-favorable environment within tissues [13]. MSCs also carry the risk of tumor or thrombus formation [14].

Nanoparticles are now being used for experimental, biomedical, and industrial purposes [15]. Extracellular vesicles (EVs) are nano-sized particles that are enclosed within lipid bilayer. They are released from numerous cell types and serve as key players for maintaining tissue homeostasis [16]. Importantly, EVs contain cargo biomolecules (e.g., RNA, protein, or lipid) that can be delivered to recipient cells, resulting in cellular homeostasis or pathological progression in a cell-free manner [17]. Therefore, the therapeutic potential of EVs derived from various cell sources are now being intensively investigated for various diseases [18]. In particular, the potential of MSC-derived EVs (MSC-EVs) for tissue repair and immune suppression has been demonstrated in various preclinical models including AD [19, 20]. In addition, quality control and long-term storage of MSC-EVs is easier than that of MSCs [21]. However, preparing large amounts of clinically applicable EVs is difficult because long-term culture as well as obtaining homogenous MSCs for EV production is technically challenging [22]. In addition, the biological profile of MSCs varies depending on the cell origin, culture method, donor age, and isolation method, resulting in inconsistencies in the cellular properties among batches [23]. Considering these drawbacks, induced MSCs (iMSCs) generated from iPSCs can be used as a novel source for producing EVs. Most importantly, a large amount of clonally derived iMSCs can be acquired, which enables the production of homogenous cells and EVs [24]. Indeed, the therapeutic function of iMSCs has been demonstrated in numerous preclinical animal models of wound healing, critical-sized bone defects, ischemia–reperfusion injuries in hindlimb, liver, and kidney, and ischemic stroke [25–30].

Importantly, the biological function of MSCs can be enhanced by preconditioning, increasing their therapeutic applicability [31]. These modified MSCs often exhibit better therapeutic potential than MSCs, mostly due to the altered secretome and biological contents of EVs. Given the unique immunosuppressive capacity of MSCs under an inflammatory environment [32], priming MSCs with pro-inflammatory cytokines is considered as an ideal option for increasing the immunomodulatory function of MSCs and the resulting EVs [33]. Interferon-γ (IFN-γ) is a major pro-inflammatory cytokine secreted from activated T lymphocytes and NK cells. It plays diverse roles in augmenting immune activity by activating the MHC and co-stimulatory molecules [34]. In MSCs, IFN-γ treatment increases the expression of IDO, TGF-β, and IL-10, all of which play critical roles in immune regulation [35, 36]. Indeed, the augmented therapeutic efficacy of IFN-γ-stimulated MSCs has been demonstrated in animal models of immune disorders such as experimental encephalomyelitis (EAE), graft-versus host disease (GvHD), and AD [34, 37–40].

MSCs are innately heterogenous, which makes its clinical use challenging because they are difficult to standardize and normalize [41]. Studies showed that iMSC closely resemble their primary counterparts in terms of shape, immunophenotype, and three-lineage differentiation capacity while demonstrating greater therapeutic efficacy in disease models [42–44]. Additionally, iPSC can be passaged indefinitely, which enables the production of homogenous cell source from single iPSC [45, 46]. Based on these aforementioned studies, herein, we assessed whether EVs produced from IFN-γ-primed iMSCs (herein, IFN-γ-iMSC-EVs) have the potential to repress AD. We generated and characterized IFN-γ-iMSC-EVs and tested their therapeutic function using 1-chloro-2,4-dinitrochlorobenzene (DNCB)-induced AD mice.

## Results

### Characterization and biodistribution of IFN-γ-iMSC-EVs

**Fig. 1:**
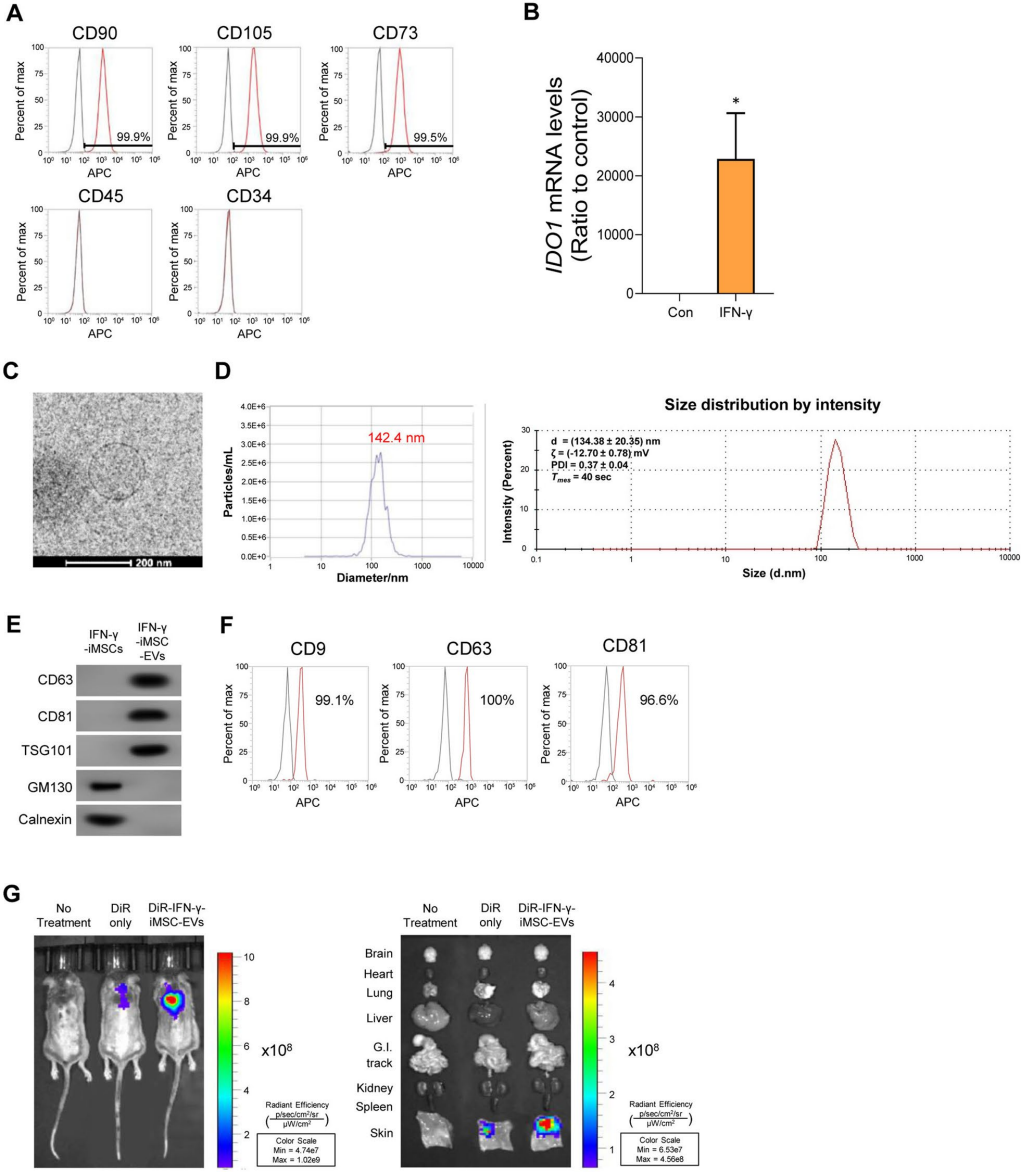
Characterization of IFN-γ-iMSCs and IFN-γ-iMSC-EVs. **A** Flow cytometry analysis of IFN-γ-iMSCs. The reactivities of IFN-γ-iMSCs against positive (CD90, CD105, and CD73) or negative (CD45 and CD34) markers of MSCs were tested. The IgG isotype was used as a non-specific control (black peaks). **B** qPCR analysis of *IDO1* mRNA in iMSCs and IFN-γ-iMSCs. *n* = 3. Data are presented as the mean ± SE. *p < 0.05. **C** Morphology of IFN-γ-iMSC-EVs under cryo-TEM. Scale bar = 200 nm. **D** Size distribution of IFN-γ-iMSC-EVs shown by NTA and DLS. The surface charge and polydispersity index (PDI) was also measured by DLS. **E** Western blot analyses for markers of extracellular vesicles (CD63, CD81, and TSG101) or cellular organelles (GM130 and calnexin) in IFN-γ-iMSCs and IFN-γ-iMSC-EVs. **F** Expression analysis of EV markers (CD9, CD63, and CD81) in IFN-γ-iMSC-EVs by flow cytometry. **G** Biodistribution of IFN-γ-iMSC-EVs. DNCB-induced AD mice were subcutaneously injected with DiR only or DiR-labeled IFN-γ-iMSC-EVs. After 8 h, the localization of DiR or DiR-labeled IFN-γ-iMSC-EVs in whole body and major internal organs was observed by in vivo imaging

IFN-γ-stimulated iMSCs were positive and negative against MSC markers (CD90, CD105, and CD73) and endothelial/hematopoietic markers (CD45 and CD34), respectively (Fig. 1A). mRNA expression of indoleamine 2,3-deoxygenase 1 (IDO1)—one of the key immune-regulatory factors induced by IFN-γ [47]—was augmented in iMSCs upon IFN-γ treatment (Fig. 1B). The average size of IFN-γ-iMSC-EVs was approximately 140 nm, as shown by the cryo-transmission electron microscopy (cryo-TEM) and nanoparticle tracking analysis (NTA) analyses. Also, Dynamic light scattering (DLS) measurement analysis showed that the diameter and zetapotential of IFN-γ-iMSC-EVs was 134.38 ± 20.35 nm and (− 12.70 ± 0.78) mV, respectively. (Fig. 1C and D). Furthermore, western blot analysis revealed that IFN-γ-iMSC-EVs expressed the typical EV markers CD63, CD81, and TSG101 (Fig. 1E), whereas they were not expressed in their parental cells, IFN-γ-stimulated iMSCs (IFN-γ-iMSCs). Similarly, flow cytometric analysis showed that IFN-γ-iMSC-EVs were positive for antibodies against CD9, CD63, and CD81, which are typical EV surface markers (Fig. 1F). In vivo tracking analyses revealed that IFN-γ-iMSC-EVs were distributed specifically in skin tissues (Fig. 1G). In vitro*,* no obvious cytotoxicity was observed in human dermal fibroblasts and keratinocytes after being exposed to IFN-γ-iMSC-EVs (Additional file 1: Fig. S1). Collectively, these results demonstrate that iMSCs maintain the expression of typical MSC markers and express immunoregulatory protein IDO in response to IFN-γ. The IFN-γ-iMSC-EVs met the minimal guidelines for EVs produced from cell supernatant, and their localization in skin tissue was confirmed [48].

### Protein signatures and pathway analysis of IFN-γ-iMSC-EVs

**Fig. 2:**
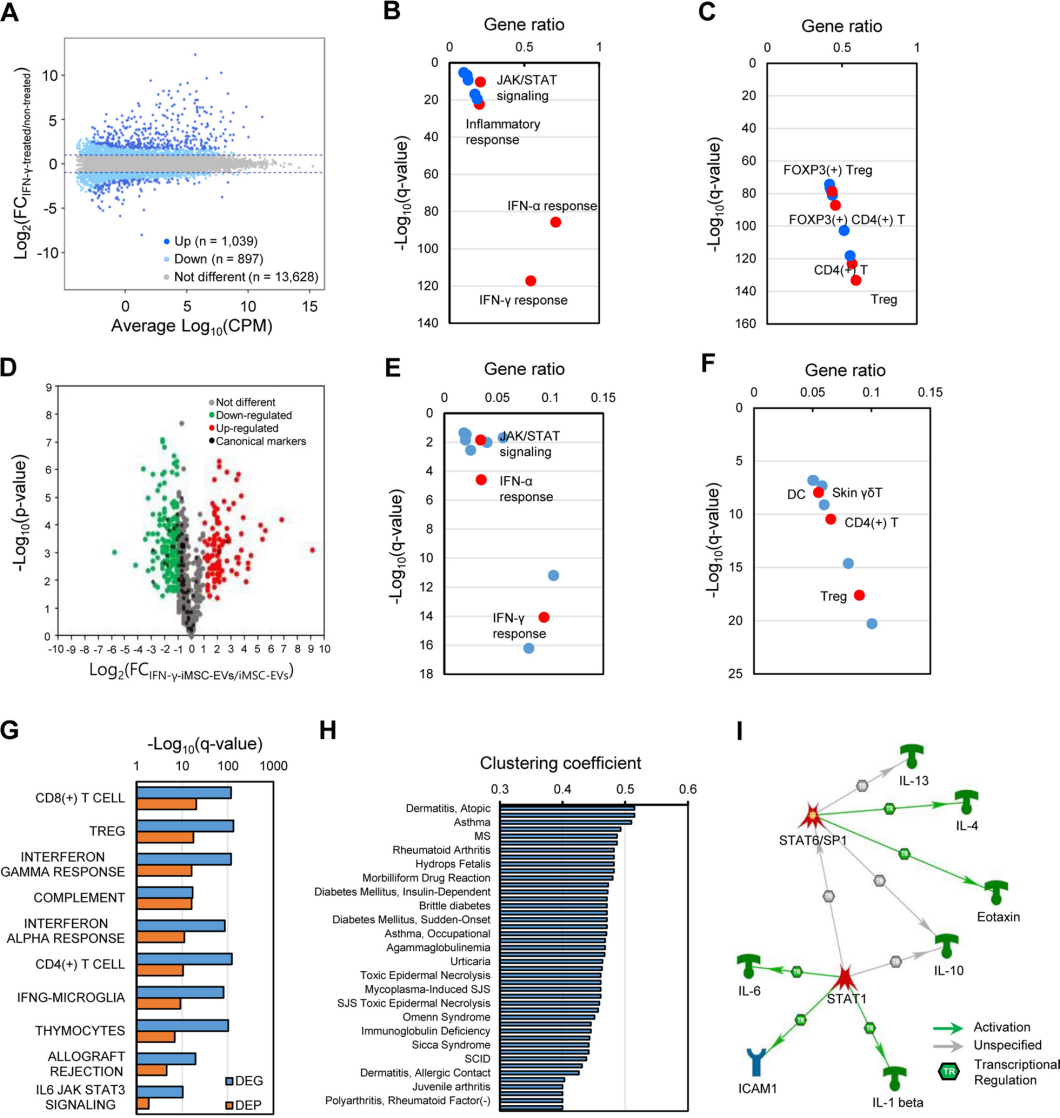
Immune system disease association with IFN-γ-iMSC-EVs. **A** IFN-γ-induced differentially expressed genes (DEGs) in iMSCs. CPM means count per million of transcripts. **B** Gene Set Enrichment Analysis (GSEA) of IFN-γ-induced up-regulated genes with hallmark collection. Red spots highlight the representative enriched functions of DEGs. **C** GSEA of IFN-γ-induced up-regulated genes with the immunologic signature gene collection. Gene ratio means the number of genes overlapping with the total number of genes in a gene set. **D** Differentially expressed proteins (DEPs) in IFN-γ-iMSC-EVs. Red circles indicate up-regulated EV proteins, green indicates down-regulated EV proteins, gray indicates non-differentially expressed EV proteins, and black indicates canonical EV markers. **E** GSEA of IFN-γ-induced up-regulated EV proteins with the hallmark collection. Red spots highlight the representative enriched functions of DEPs. **F** GSEA of IFN-γ-induced up-regulated EV proteins with the immunologic signature gene collection. **G** Comparison between the enriched functions of IFN-γ-induced DEGs and EV DEPs. Blue bars indicate the enriched functions of DEGs and red bars indicate DEPs. **H** Clustering coefficient with the IFN-γ-induced EV DEPs and immune system disease gene set. The horizontal axis indicates the local clustering coefficient and vertical axis indicates the representative immune system diseases associated with DEPs of IFN-γ-iMSC-EVs over iMSC-EVs. **I** Core protein–protein interaction network constructed with the atopic dermatitis gene set and DEPs of IFN-γ-iMSC-EVs over iMSC-EVs. Green arrows indicate transcriptional activation and gray unspecified effects

IFN-γ treatment induced significant changes in the transcription profile in iMSCs, resulting in 1039 up-regulated genes and 897 down-regulated genes (Fig. 2A). Among them, up-regulated genes were subjected to gene set enrichment analysis (GSEA). IFN-γ treatment significantly up-regulated the genes involved in IFN-γ response (q = 5.67 × 10^–118^), IFN-α response (q = 1.55 × 10^–86^), inflammatory response (q = 3.50 × 10^–23^), and the JAK–STAT signaling pathway (q = 4.21 × 10^–11^) (Fig. 2B and Additional file 2: Table S1). GSEA of IFN-γ-induced genes also revealed that they are involved in Treg cell activation (q = 4.63 × 10^–134^) and T-cell activation (q = 7.26 × 10^–124^) (Fig. 2C and Additional file 2: Table S2). These transcriptional changes were faithfully reflected in EVs released by IFN-γ-treated iMSCs as well. Proteomic analysis of EV proteins demonstrated that 101 proteins were significantly up-regulated and 181 proteins were significantly down-regulated by IFN-γ treatment. Furthermore, 25 proteins were exclusively identified in IFN-γ-induced EVs (Fig. 2D). Among them, up-regulated proteins were subjected to GSEA, and proteins up-regulated in IFN-γ-induced EVs were significantly enriched in the Hallmark and immunological signatures comparable to IFN-γ-induced differentially expressed genes (DEGs) (Fig. 2E and F). For example, the up-regulated EV proteins were significantly enriched in IFN-γ response (q = 6.23 × 10^–17^), the JAK-STAT signaling pathway (q = 1.39 × 10^–2^), T-cell activation (q = 5.21 × 10^–21^), and Treg-cell activation (q = 2.40 × 10^–18^) (Fig. 2G, Additional file 2: Tables S3 and S4). These results showed that the molecular and biological changes of iMSCs by IFN-γ would lead to changes in the composition of EV protein cargo for immunological application. To discover more promising indicators of the modified EVs, gene sets of various immune system diseases were collected, and the association between the disease signatures and IFN-γ-induced EV proteins was analyzed using clustering coefficient estimation. Among the immune system diseases, atopic dermatitis (clustering coefficient = 0.515) and infantile eczema (clustering coefficient = 0.515) were identified as the top associated immune diseases with IFN-γ-induced EV proteins (Fig. 2H and Additional file 2: Table S5). Core network analysis also suggested that STAT1 from IFN-γ-induced EVs played a key role in regulating the essential signaling pathway of STAT6-mediated atopy (Fig. 2I). Therefore, these results suggest the possibility that EVs modified by IFN-γ treatment would be effective against atopy among various immune system diseases.

### In vivo assessment of IFN-γ-iMSC-EV function in AD

**Fig. 3:**
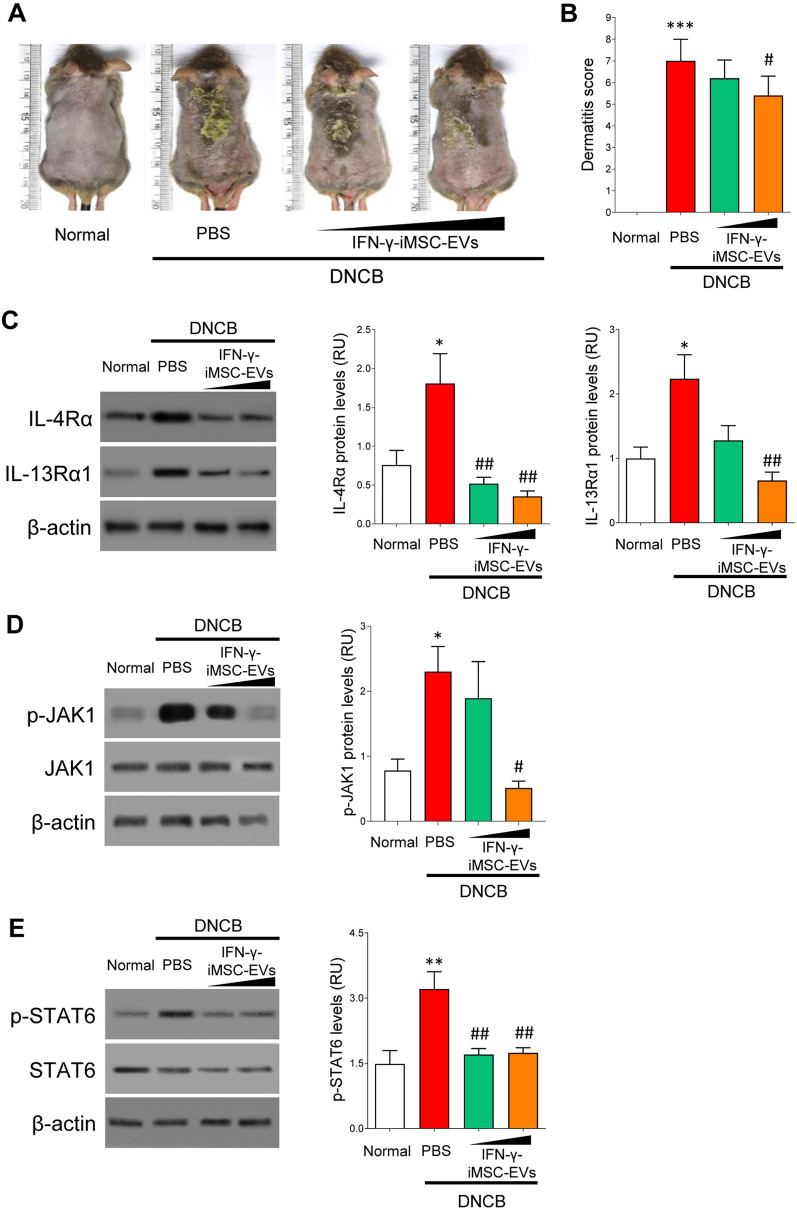
Blockade of AD progression by IFN-γ-iMSC-EVs. AD was induced by DNCB in NC/Nga mice. AD mice were subcutaneously administered with PBS or IFN-γ-iMSC-EVs (50 or 500 μg) for negative control and test groups, respectively. **A** Gross appearance of the skin lesions of AD animals that received IFN-γ-iMSC-EVs. **B** Analysis of dermatitis severity score. *n* = 5. Data are presented as mean ± SE. ***p < 0.001; ^#^*p* < 0.05. **C** Immunoblot analysis of IL-4Rα and IL-13Rα1 in skin tissues from AD mice that received IFN-γ-iMSC-EVs. *n* = 5. Data are presented as mean ± SE. *p < 0.05; ^##^*p* < 0.01. **D** Immunoblot analysis of phosphorylated JAK1 in skin tissues from AD mice that received IFN-γ-iMSC-EVs. The density of phosphorylated JAK1 was normalized to that of total JAK1. *n* = 5. Data are presented as the mean ± SE. *p < 0.05; ^#^*p* < 0.05. **E** Immunoblot analysis of phosphorylated STAT6 in skin tissues from AD mice that received IFN-γ-iMSC-EVs. The density of phosphorylated STAT6 was normalized to that of total STAT6. *n* = 5. Data are presented as mean ± SE. **p < 0.01; ^##^*p* < 0.01

THE therapeutic function of IFN-γ-iMSC-EVs was examined using DNCB-induced AD in NC/Nga mice. No difference was observed in the lethality and body weight between AD mice treated with PBS vs. IFN-γ-iMSC-EVs, suggesting that IFN-γ-iMSC-EVs does not induce adverse effect in animals (Additional file 1: Fig. S2). As shown in Fig. 3A, AD-like skin lesions were observed in DNCB-treated mice. In contrast, AD-like skin lesions were less severe in animals treated with IFN-γ-iMSC-EVs (Fig. 3A), which reduced the overall dermatitis score (Fig. 3B). Immunoblot analysis showed that the expression of IL-4Rα and IL-13Rα1 proteins was decreased by IFN-γ-iMSC-EVs in a dose-dependent manner compared to their expression induced by PBS (Fig. 3C). JAK–STAT is the key downstream pathway of IL-4Rα and IL-13Rα1, which has been known to play a critical role in the regulation of immune responses in AD [49]. Immunoblot analysis revealed that the phosphorylation of JAK1 and STAT6 was decreased by IFN-γ-iMSC-EVs compared to vehicle (PBS) treatment (Fig. 3D and E). Collectively, these data indicate that IFN-γ-iMSC-EVs can suppress AD progression by inhibiting the expression of IL-4Rα and IL-13Rα1.

### Attenuation of skin inflammation by IFN-γ-iMSC-EVs

**Fig. 4:**
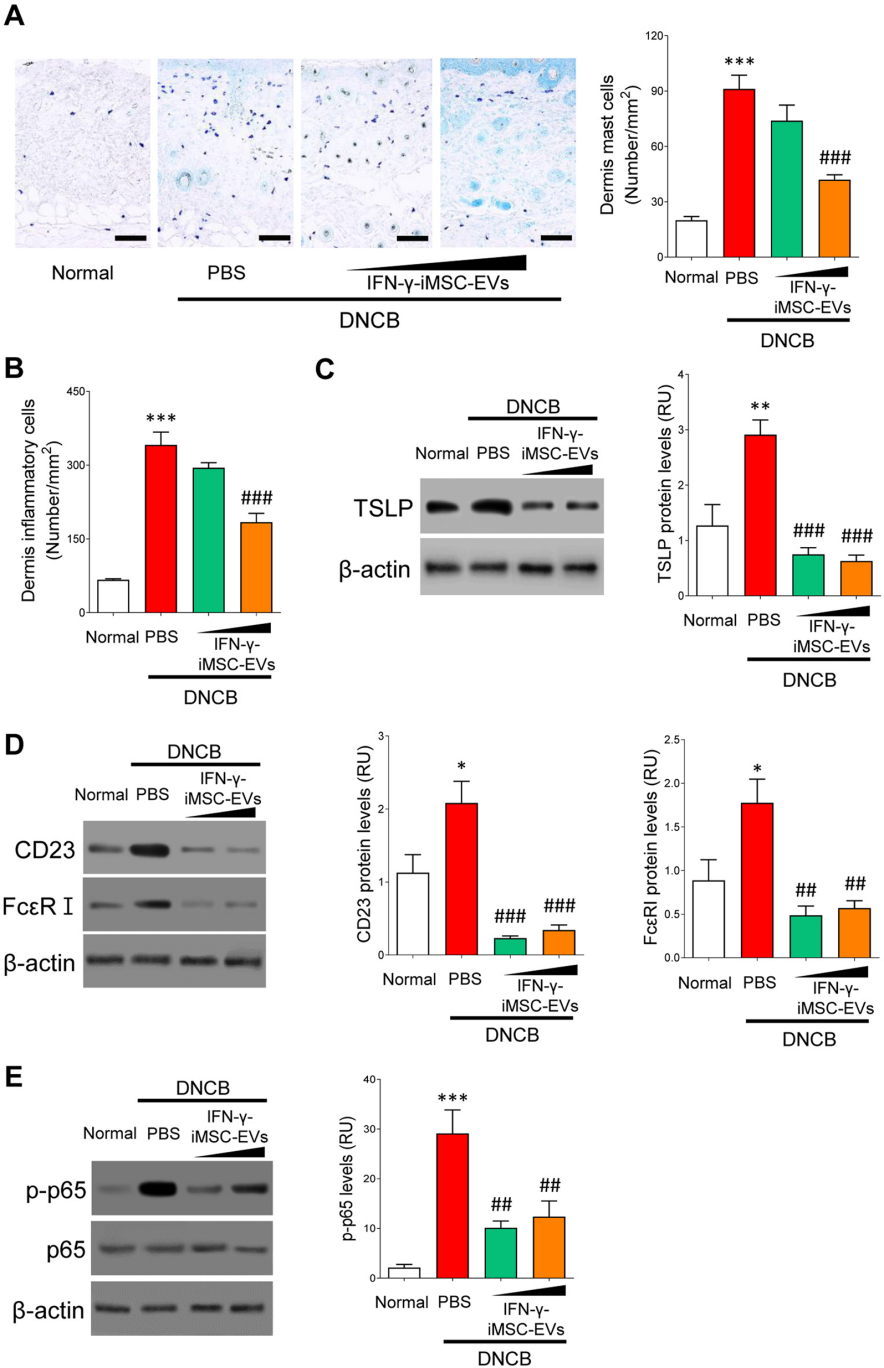
Attenuation of inflammation by IFN-γ-iMSC-EVs in AD mice. AD mice were subcutaneously administered with PBS or IFN-γ-iMSC-EVs (50 or 500 μg) for negative control and test groups, respectively. **A** Distribution and number of mast cells in skin tissues. Mast cells in the skin layer were examined by staining with Toluidine blue. Scale bar: 100 μm. *n* = 5. Data are presented as mean ± SE. ***p < 0.001; ^###^*p* < 0.001. **B** Analysis of inflammatory cell number in the skin layer of AD mice that received PBS or IFN-γ-iMSC-EVs. *n* = 5. Data are presented as mean ± SE. ***p < 0.001; ^###^*p* < 0.001. **C** Immunoblot analysis of TSLP in the skin tissues collected from AD mice that received PBS or IFN-γ-iMSC-EVs. *n* = 5. Data are presented as mean ± SE. **p < 0.01; ^###^*p* < 0.001. **D** Immunoblot analysis of CD23 and FcεRI in skin tissues of AD mice that received PBS or IFN-γ-iMSC-EVs. *n* = 5. Data are presented as mean ± SE. *p < 0.05; ^##^*p* < 0.01; ^###^*p* < 0.001. **E** Immunoblot analysis of phosphorylated p65 in skin tissues from AD mice that received PBS or IFN-γ-iMSC-EVs. The density of phosphorylated p65 was normalized to that of total p65. *n* = 5. Data are presented as mean ± SE. ***p < 0.001; ^##^*p* < 0.01

AD is a chronic and multifactorial inflammatory skin disease [50]. We evaluated the anti-inflammatory effects of IFN-γ-iMSC-EVs in AD. The number of mast cells and inflammatory cells in the dermis were markedly increased in AD mice. In contrast, IFN-γ-iMSC-EVs potently decreased the number of these cells in a dose-dependent manner (Fig. 4A and B). The skin tissue of AD mice showed an increased level of TSLP, which is a critical proinflammatory cytokine responsible for inflammation in AD [51]. Consistently, IFN-γ-iMSC-EVs markedly downregulated TSLP expression compared with vehicle (PBS)-treated animals (Fig. 4C). CD23 and FcεRI are high-affinity receptors for IgE that are observed in mast cells and basophiles, which control inflammation in AD [52]. Thus, we examined the expression of both receptors by immunoblot analysis. Protein expression of CD23 and FcεRI was increased in AD mice, which was decreased by IFN-γ-iMSC-EVs compared to those cells that received PBS (Fig. 4D). Additionally, the increase of p65 activation in AD skin tissue was reversed by IFN-γ-iMSC-EVs compared to PBS treatment (Fig. 4E). Altogether, these data indicate that IFN-γ-iMSC-EVs ameliorate skin inflammation by inhibiting the proliferation of immune cells and blocking TSLP expression, all of which might be involved in the reduced activity of the NF-κB pathway.

### Alleviation of pruritus by IFN-γ-iMSC-EVs

**Fig. 5:**
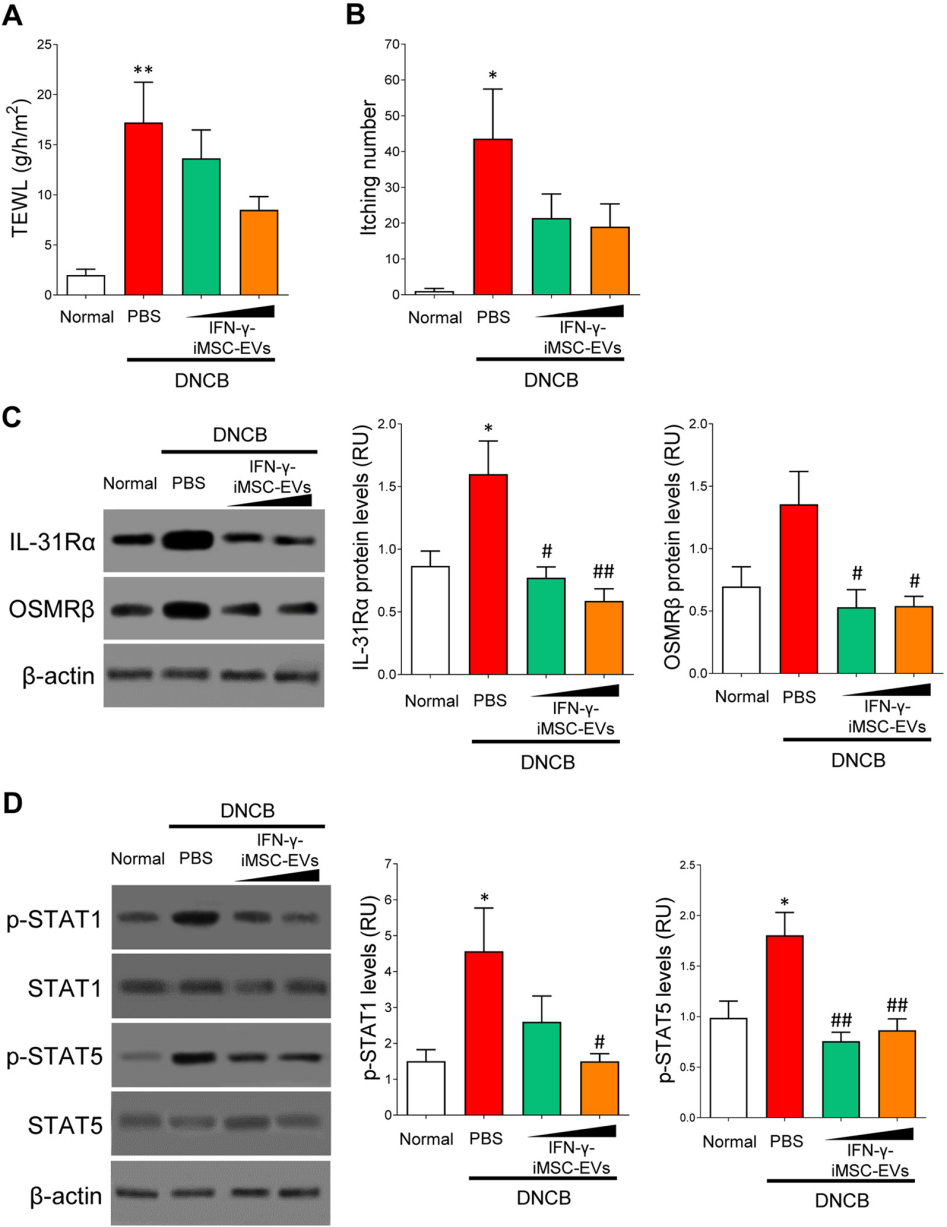
Reduction of pruritus by IFN-γ-iMSC-EVs in AD mice. AD mice were subcutaneously administered with PBS or IFN-γ-iMSC-EVs (50 or 500 μg) for the negative control and test groups, respectively. **A** Analysis of transepidermal water loss (TEWL) levels. *n* = 5. Data are presented as mean ± SE. *p < 0.05; **p < 0.01. **B** The effect of IFN-γ-iMSC-EVs on itching number in AD mice. *n* = 5. Data are presented as mean ± SE. *p < 0.05. **C** Immunoblotting of IL-31Rα and OSMRβ in the skin tissue of AD mice that received IFN-γ-iMSC-EVs. *n* = 5. Data are presented as mean ± SE. *p < 0.05; ^#^P < 0.05; ^##^*p* < 0.01. **D** Immunoblot analysis of phosphorylated STAT1 and STAT5 expression in skin tissues of AD mice that received IFN-γ-iMSC-EVs. Densities of phosphorylated STAT1 and STAT5 were normalized to those of total STAT1 and STAT5, respectively. *n* = 5. Data are presented as mean ± SE. *p < 0.05; ^#^P < 0.05; ^##^*p* < 0.01

Pruritus is one of the major symptoms of AD accompanied loss of hydration [53, 54]. Figure 5A shows that transepidermal water loss (TEWL) was increased in AD skin, which was reversed by IFN-γ-iMSC-EVs (Fig. 5A). Consistently, similar findings were observed for the effect of IFN-γ-iMSC-EVs on itching number (Fig. 5B). IL-31 is a dominant pruritic cytokine secreted from Th2 cells in AD skin, and activation of receptors of IL-31 is directly associated with pruritic disease [55]. Immunoblot analysis revealed that the expression of IL-31Rα and OSMRβ proteins was decreased by IFN-γ-iMSC-EVs than by PBS (Fig. 5C). Additionally, the activation of STAT1 and STAT5 signaling was suppressed by IFN-γ-iMSC-EVs (Fig. 5D). These data support that IFN-γ-iMSC-EVs block the pruritus by suppressing IL-31R-STAT signaling.

### Amelioration of skin barrier and lipid synthesis by IFN-γ-iMSC-EVs

**Fig. 6:**
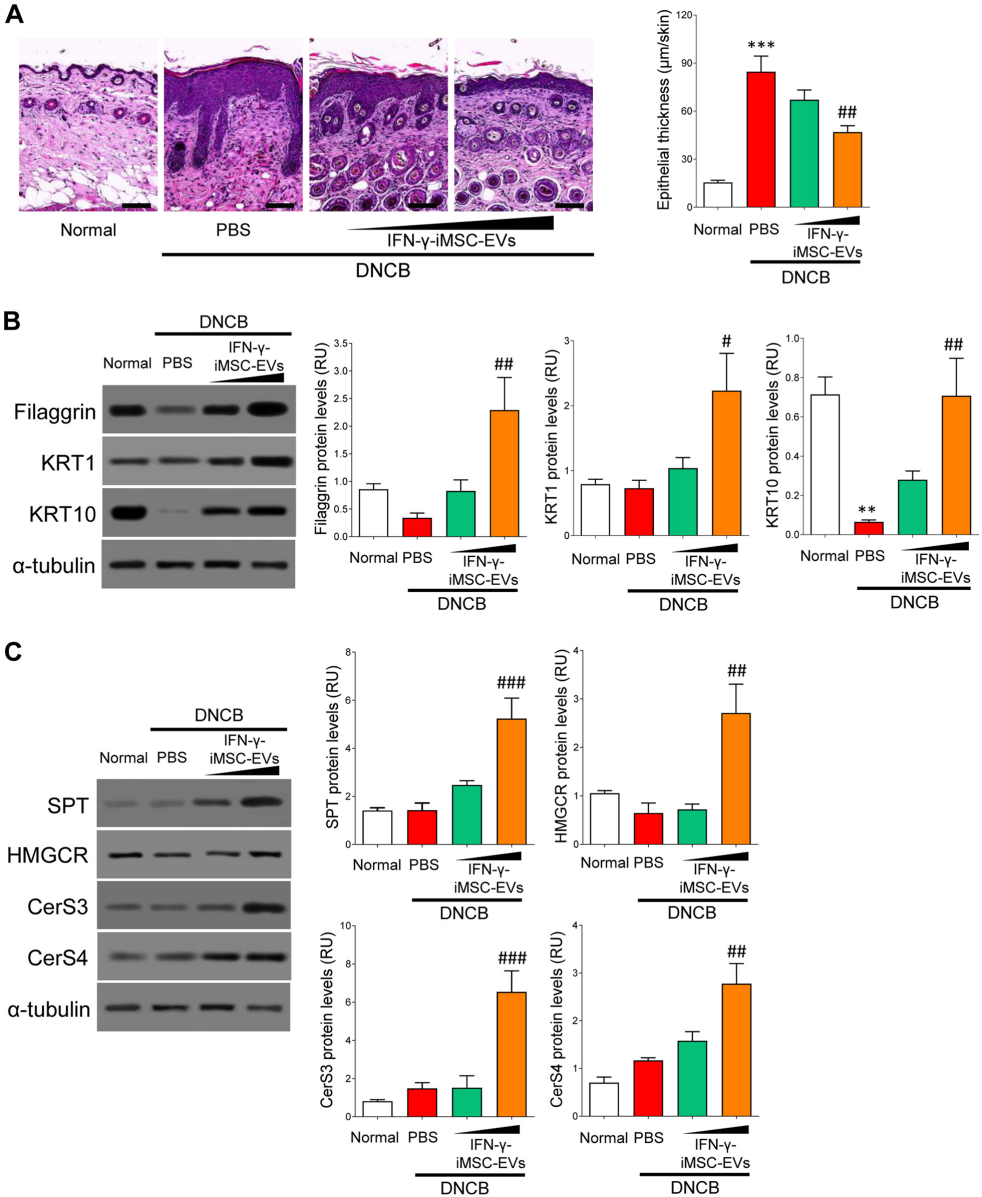
Restoration of skin barrier and lipid synthesis by IFN-γ-iMSC-EVs in the epidermis of AD mice. AD mice were subcutaneously administered with PBS or IFN-γ-iMSC-EVs (50 or 500 μg) for negative control and test groups, respectively. **A** Microscopic images of the skin tissues collected from IFN-γ-iMSC-EVs-injected AD mice. Tissues were stained with hematoxylin and eosin. The thickness of the epithelium was compared. Scale bar: 100 μm. *n* = 5. Data are presented as mean ± SE. ***p < 0.001; ^##^*p* < 0.01. **B** Immunoblot analysis of skin barrier-related proteins in the epidermis of AD mice that received PBS or IFN-γ-iMSC-EVs. *n* = 5. Data are presented as mean ± SE. **p < 0.01; ^#^*p* < 0.05; ^##^*p* < 0.01. **C** Immunoblot analysis of proteins involved in lipid synthesis in the epidermal tissue of AD mice that received PBS or IFN-γ-iMSC-EVs. *n* = 5. Data are presented as mean ± SE. ^##^*p* < 0.01; ^###^*p* < 0.001

The skin of AD is characterized by impaired barrier function and lipid abnormalities [56, 57]. Histological analysis showed that the epithelial thickness was increased in AD skin, which was then decreased by high doses of IFN-γ-iMSC-EVs compared to vehicle control (PBS) (Fig. 6A). Consistently, the expression of genes responsible for skin barrier integrity (Filaggrin, Keratin 1 (KRT1), and Keratin 10 (KRT10)) was increased by 500 μg of IFN-γ-iMSC-EVs (Fig. 6B). Furthermore, the levels of lipid synthesis-related proteins (serine palmitoyltransferase (SPT), HMG-CoA reductase (HMGCR), ceramide synthase 3 (CerS3), ceramide synthase 4 (CerS4) was enhanced by high doses of IFN-γ-iMSC-EVs (Fig. 6C). Collectively, these data demonstrate that IFN-γ-iMSC-EVs can restore AD-induced skin barrier dysfunction and abnormal lipid synthesis.

## Discussion

In this study, we show that IFN-γ-iMSC-EVs repress AD primarily by inhibiting the expression of Th2 cytokine receptors (i.e., IL-4Rα, IL-13Rα1, and IL-31Rα) and their downstream signaling mediators. We confirmed that IFN-γ-iMSC-EVs meet the guidelines for EVs as characterized by the morphology, size distribution, and expression of typical protein markers for EVs [48, 58]. Biodistribution study revealed that IFN-γ-iMSC-EVs are specifically located in the skin tissue, indicating that they are compatible with the local SC administration protocol for AD. To determine the molecular function of IFN-γ-iMSC-EVs, we identified IFN-γ-responsive DEGs in iMSCs. IFN-γ-iMSCs was enriched with key inflammatory pathways including the JAK/STAT and IFN pathways. Consistently, subsequent proteome profiling and bioinformatic analysis of upregulated proteins in IFN-γ-iMSC-EVs showed that they share enriched pathways with those from IFN-γ-iMSC. Moreover, IFN-γ-iMSC-EVs expressed proteins involved in T-cell function and regulation. Furthermore, network analysis showed that proteins of IFN-γ-iMSC-EVs are involved in atopic dermatitis, asthma, and other immunological diseases. In vivo, IFN-γ-iMSC-EVs markedly reduced the expression of IL-4α/13Rα1 and inhibited the activation of JAK1 and STAT6 in the AD skin tissue. IFN-γ-iMSC-EVs also reduced the proliferation of mast cells, expression of IgE receptors (CD23, FcεR1), NF-κB, TSLP, all of which are contributors of AD. IFN-γ-iMSC-EVs also decreased the expression of IL-31Rα and OSMRβ, as well as their downstream players (STAT1/5), reducing pruritus and itching. Finally, IFN-γ-iMSC-EVs enhanced skin barrier integrity, as shown by the increase in the epithelial thickness and augmented expression of genes responsible for terminal epithelial differentiation and lipid synthesis in AD skin. Collectively, our data suggest that IFN-γ-iMSC-EVs have the potential to inhibit AD by playing an anti-inflammatory and immunoregulatory role.

Among various Th2 responses in allergic diseases, IL-4/13 are the most well-characterized players [4]. Previous study showed that IL-4Rα and 13Rα1 are expressed in the primary sensory neurons of dorsal root ganglion that innervates dermal tissue, thereby enabling neuronal activation by IL-4/13 [59, 60]. Together with other pruritic cytokines IL-31 and TSLP, this response increases the susceptibility to itching, creating a vicious “itch-scratch” cycle [2]. IFN-γ-iMSC-EVs could reduce itching, which is likely due to the reduced expression of multiple players in AD progression.

Various drugs including anti-histamines, anti-leukotrienes, and corticosteroids have been used to reduce the extreme immune responses in AD lesions [3]. However, repeated use of these drugs can cause side effects [61]. Dupilumab, a monoclonal antibody that targets IL-4Rα and subsequent IL-4/13 signaling, was approved by the US FDA in 2017 and is recognized as a safe option for long-term use in moderate-to-severe AD. However, complete skin clearness was observed in only less than 40% of patients that received dupilumab [62]. In addition, the incidence of ocular complication associated with dupilumab reached 43–70%, depending on the analysis method and patient characteristics [63–65]. Furthermore, dupilumab can predispose patients to developing to chronic stages of the disease, which requires immunosuppressive treatment through steroids or calcineurin inhibitors [63]. Thus, other treatment strategies for AD that can target multiple players with better immune-compatibility and safety are required. Indeed, biologics that target IL-13 (tralokinumab), as well as those that antagonize JAK1 (abrocitinib) and JAK1/2 (ruxolitinib), have recently been approved for clinical use [6]. Although optimistic outcomes have been observed for reducing pruritus and inflammation, long-term data are lacking, and their benefit-to-risk ratio should be further improved [6]. Moreover, these drugs have been developed for single or dual targets, necessitating the development of novel therapeutics that can inhibit multiple pathways of AD. Notably, treatment strategies of AD should be determined based on the ruling mechanism as well as severity, mostly to reduce pruritus, pain, and skin lesions. In this regard, IFN-γ-iMSC-EVs, which is armed with immune-regulatory function, would be an ideal agent that can be used independently or with other drugs (Table 1).

**Table 1:** Comparison of current therapeutic strategy for atopic dermatitis

Drug name (Commercial name)	Technology	Mechanism	Administration route	Development status	References
Crisaborole (Eucrisa)	Small molecule (Topical)	Phosphodiesterase inhibitor	Topical	Approved (US, 2016)	[81]
Dermavant (Tapinarof)	Small molecule (Topical)	Targets AhR agonist, thus blocking innate immunity	Topical	Approved (US, 2022)	[82]
Abrocitini (Cibinqo)	Small molecule	JAK1 inhibitor	Oral	Approved (EU, US, 2021)	[83]
Upadacitinib (Rinvoq)	Small molecule	JAK1 inhibitor	Oral	Launched (EU/US, 2021)	[84]
Delgocitinib (Corectim)	Small molecule	Pan-JAK inhibitor	Topical	IIb (EU) Approved (Japan, 2021)	[85]
Dupilumab (Dupixent)	Monoclonal antibody	Binds to IL-4Rα, thus blocking IL-4 and IL-13 signaling	SC injection	Launched (EU/US, 2017)	[86]
Tralokinumab Adbry (US) Adtralza (EU/UK)	Monoclonal antibody	Binds to IL-13	SC injection	Approved (EU/US, 2021)	[87]
Nemolizumab (Mitchga^®^ Syringe)	Monoclonal antibody	Targets IL-31Rα, thus blocking itching	SC injection	Approved (Japan, 2022) Phase III (EU/US)	[88]
Ruxolitinib (Opzelura)	Monoclonal antibody	Dual JAK1/JAK2 inhibitor	Topical	Launched (US, 2021) Phase III (EU)	[89]
BRE-AD01	EV	Targets IL-4Rα, IL-13Rα1 and IL-31Rα Skin tissue regeneration	SC injection	Phase 1 (US, 2022)	NA

*AhR* aryl hydrocarbon receptor, *EV* extracellular vesicle, *IL-4Rα* interleukin-4 receptor alpha subunit, *IL-31Rα* interleukin-31 receptor alpha subunit, *JAK* Janus kinase, *NA* not applicable, *SC* subcutaneous

Given the immunoregulatory and anti-inflammatory function [19], EVs from MSCs could be an alternative that can replace the current therapeutic protocol for AD [66]. Indeed, it was demonstrated that EVs from adipose-derived stem cells (ADSCs) could alleviate AD by reducing serum IgE, infiltration of inflammatory cells, and inflammatory cytokines. Furthermore, restoration of epidermal barrier function was induced by upregulating skin lipid synthesis, all of which contributed to repressing AD [67, 68]. However, these studies lack information regarding how ADSC-EVs block AD progression or which pathological players are affected. We, for the first time, show that iMSC-derived EVs can specifically block the expression of key signaling receptors IL-4Rα/13Rα1 as well as their downstream mediators JAK1 and STAT6. Consistently, IFN-γ-iMSC-EVs blocked the expression of IL-31Rα and OSMRβ, as well as their signaling mediators STAT1/5, all of which might have contributed to AD inhibition, as demonstrated by reduced inflammation and recovery of skin barrier function and epithelial integrity. Detailed molecular mechanisms through which IFN-γ-iMSC-EVs negatively regulate interleukin receptors are needed for better understanding of the therapeutic action of IFN-γ-iMSC-EVs in AD.

MSCs interact with various lymphocytes and exert an immune regulatory role by secreting soluble factors and EVs [40, 69, 70]. Importantly, the immunomodulatory or tissue-reparative function of MSC-derived EVs can be largely affected by treatment with cytokines or growth factors [71]. IFN-γ is a soluble cytokine that belongs to the type II class of interferon, which is produced from various cells including T-cells, NK T cells, NK cells, macrophages [72]. Upon binding to IFN-γ receptor 1 and 2, the JAK–STAT1 pathway is activated, promoting the expression of IDO1. This IFN-γ-IDO1 axis is responsible for the immunomodulatory function of MSCs [73]. IDO1 degrades the essential amino acid tryptophan, resulting in the generation of metabolites called “kynurenines”. Subsequently, these metabolites induce apoptotic cell death of T-cells [74] and also contribute to an increase in immunoregulatory T cells [75]. Other studies also demonstrated that kynurenines suppress the apoptosis of other immune cells including T, B, and natural killer cells [73, 76, 77]. Indeed, the enhanced immunomodulatory function of MSCs by IFN-γ was demonstrated in *Aspergillus fumigatus*-induced AD; IFN-γ-stimulated MSCs led to a decrease in epidermal thickness and inflammatory cell deposition in skin compared with treatment with non-treated MSCs [78]. Thus, it can be suggested that the immune regulatory function of MSCs induced by IFN-γ is largely dependent on their EVs. To address this, the therapeutic mechanism and function of IFN-γ-iMSC and IFN-γ-iMSC-EVs must be compared in AD.

## Conclusions

In conclusion, our data show that IFN-γ-iMSC-EVs reduce AD by inhibiting the expression of Th2 cytokine receptors and their downstream signaling mediators. Together with its immune-regulatory function, IFN-γ-iMSC-EVs substantially restored epidermal barrier function and lipid synthesis in AD skin. The results of our study may contribute to developing novel cell-free therapeutic strategies for AD.

## Materials and methods

### Animals

Six-week-old specific pathogen free NC/Nga male mice were obtained from SLC Inc. (Hamamatsu, Japan). Animal care and procedures were approved by Institutional Animal Care and Use Committee (IACUC) of Chemon (Korea; Serial Number: 2021-07-018) and Seoul National University (#SNU-210927-5). After acclimation, NC/Nga mice were anesthetized using isoflurane, and their dorsal hair was shaved. After 24 h, 200 μL of 1% DNCB in acetone/olive oil (3:1) was topically applied to the dorsal skin, twice a week for sensitization. Thereafter, 150 μL of 0.4% DNCB in acetone/olive oil (3:1) was topically applied to challenge the dorsal skin, 3 times a week for 5 weeks. From 2 weeks after the first application, 50 or 500 μg of IFN-γ-iMSC-EVs, respectively, were subcutaneously injected once a week for 5 weeks.

### Analysis of atopic dermatitis score

The atopic dermatitis scores were graded as follows: none (0), mild (1), moderate (2), and severe (3) for each of the following five symptoms for the evaluation index: erythema, dry skin, edema and hematoma, erosion, and lichenification. A total dermatitis score was defined as the sum of all scores (maximum score: 15). The evaluation was performed at the same time once a week.

### Measurement of itching behavior and transepidermal water loss

To evaluate scratching behavior, the scratching frequency was assessed over 30 min. One scratch was considered to be the lifting of the hind limb toward the area and then replacing of the limb back to the floor or licking the hind limb. Transepidermal water loss (TEWL) was measured on the dorsal skin before necropsy by GPSKIN Barrier Research Solution-I (Gpskin, Gyeonggi, South Korea).

### Epidermis isolation

The subcutaneous fat was removed from a skin sample using a scalpel, and the skin sample was incubated in 10 mM ethylenediamine tetraacetic acid (EDTA) in phosphate-buffered saline (PBS) at 37 °C for 35 min. Then, epidermis was scraped off with a scalpel [79].

### Isolation of IFN-γ-iMSC-EVs

IFN-γ-stimulated iMSCs were replaced with serum-free and xeno-free medium (RoosterBio, Frederick, MD, USA). After 24 h of incubation, the culture medium was harvested and centrifuged for 10 min at 300×*g*, and the supernatant was centrifuged for 20 min at 2000×*g*. The supernatant was centrifuged for an additional 80 min at 10,000×*g*. Thereafter, the supernatant was filtered through a 0.2 μm vacuum filter (Merck Millipore, Burlington, MA, USA). Lastly, IFN-γ-iMSC-EVs were isolated using ultracentrifugation (Beckman Coulter, CA, USA) at 100,000×*g* for 80 min, and the pellet was subsequently washed with PBS and collected by ultracentrifugation at 100,000×*g* for 80 min. The IFN-γ-iMSC-EVs pellets were resuspended in PBS.

### Cryo-TEM

A 200-mesh copper grid (MiTeGen, Ithaca, NY, USA) coated with formvar/carbon film was subjected to hydrophilic treatment. The IFN-γ-iMSC-EV suspension (4 μL) was placed on a grid and blotted for 1.5 min at 100% humidity and 4 °C. The IFN-γ-iMSC-EVs on the grid were visualized at a magnification of 36,000× using a Talos L120C FEI transmission electron microscope (Thermo Fisher Scientific) at 120 kV.

### Nanoparticle tracking analysis (NTA) assay

Measurements of particle size distribution and concentration of IFN-γ-iMSC-EVs were performed using the ZetaView Nanoparticle Tracking Analyzer PMX-120 instrument (Particle Metrix, Inning am Ammersee, Germany) based on NTA. For analyses, IFN-γ-iMSC-EVs were diluted in sterile PBS to reach the optimal volume for NTA. Measurements were performed at room temperature ranging from 23.0–25.2 °C using a 488 nm laser and the high sensitive CMOS camera in several repeats. Sample analysis was conducted under the following camera settings and processing conditions: sensitivity 80, shutter 100, 2 cycles, 11 positions, NTA software version 8.05.14_SP7.

### Dynamic light scattering (DLS) and surface potential measurement

The size distribution, zeta-potential, and polydispersity index of IFN-γ-iMSC-EVs was analyzed with a Zetasizer Nano-ZS and the software Zetasizer version 7.12 (Malvern Panalytical Ltd, UK). IFN-γ-iMSC-EVs (3 μg/mL) were diluted in PBS and measurement was conducted for three replicates at room temperature according the the manufacturer’s instruction.

### Flow cytometry

IFN-γ-iMSC-EVs were stained using the human MACSPlex Exosome Kit (Miltenyi Biotec, Bergisch Gladbach, Germany) and analyzed using an Attune NxT flow cytometer (Thermo Fisher Scientific). To confirm whether IFN-γ-stimulated iMSCs express the typical cell surface markers for MSCs, IFN-γ-stimulated iMSCs were stained with CD73 APC, CD105 PE, CD45 FITC, and CD34 APC (eBioscience, Waltham, MA, USA) and CD90 APC-Cy7 (BioLegend) antibodies. Flow cytometric analysis was performed using the Attune NxT flow cytometer (Thermo Fisher Scientific).

### Fluorescent imaging IFN-γ-iMSC-EVs

IFN-γ-iMSC-EVs were incubated with 1 μg/mL DiR buffer for 10 min at 37 °C according to the protocol mentioned by Lipophilic Tracers (Invitrogen, Waltham, MA, USA). Next, the DiR-labeled IFN-γ-iMSC-EVs were centrifuged for 80 min at 100,000×*g* and 4 °C and washed with PBS (Gibco). Lastly, 100 μg of DiR-labeled IFN-γ-iMSC-EVs were resuspended in 0.2 mL of PBS and subcutaneously injected into DNCB-induced NC/Nga mice. After 8 h, DiR-labeled IFN-γ-iMSC-EVs were detected using an In Vivo Imaging System (Caliper Life Sciences, Waltham, MA, USA) at excitation and emission wavelengths of 740 and 790 nm, respectively. The intensity of the region of interest was plotted in units of the maximum number of photons per second per centimeter square per steradian (p/s/cm^2^/sr).

### Bioinformatic analyses

iMSCs were treated with IFN-γ at 20 ng/mL for 24 h, and the total RNA was isolated using the RNeasy Mini Kit (Qiagen, Hilden, Germany). RNA expression levels were measured using RNA-sequencing (Illumina, San Diego, CA, USA), and the IFN-γ-induced DEGs were identified with the fold change (FC) cutoff = 2. EVs were isolated from iMSCs stimulated with IFN-γ for 24 h. EV proteins were extracted and subjected to the reversed-phase fractionation liquid chromatography (Agilent Technologies, Santa Clara, CA, USA) followed by mass spectrometry (Thermo Fisher Scientific, Waltham, MA, USA). IFN-γ-induced differentially expressed EV proteins (DEPs) were identified with the FC cutoff = 2 and the p-value cutoff = 0.05. The DEGs and DEPs were subjected to GSEA with Hallmark and immunological signature collections at false discovery rate (FDR) q-value cutoff = 0.05 (http://www.gsea-msigdb.org/gsea). The upregulated EV proteins using IFN-γ treatment were subjected to gene-disease association analysis. Gene signatures of 44 immune system diseases were collected from the curated resources in DisGeNET (https://www.disgenet.org/) and were subjected to estimate the local clustering coefficient with the IFN-γ-induced EV proteins using functional protein association network analysis (https://string-db.org/). The core protein–protein interaction network between the disease signature and the EV proteins was identified and visualized using MetaCore (https://portal.genego.com/). Total proteins were identified with 1.0% of FDR (false discovery rate). A label-free quantification was performed with 3 repetitive sample processing and LC–MS/MS measurements. The quantitative values of the identified proteins for each sample were normalized for a comparative analysis. Then, identified peptides with coefficient variation (CV, %) values of less than 30% were quantified.

### Real-time qPCR

For measurement of mRNA expression, total RNA was isolated, and real-time qPCR was performed, as described previously [80]. The primer sequences are listed in Additional file 1: Table S7.

### Western blot analysis

Skin tissues were lysed in RIPA lysis buffer (Thermo Fisher Scientific) supplemented with protease and phosphatase inhibitors (Thermo Fisher Scientific). The protein concentration was measured using the Bradford Assay™ Reagent (Thermo Fisher Scientific) according to the manufacturer’s protocol. Samples were diluted in 3:1 ratio using the 4× Laemmli buffer (Bio-Rad Laboratories, Hercules, CA, USA) and heated to 100 °C for 10 min. Proteins were loaded and separated on precast polyacrylamide Mini-PROTEAN TGX gels (Bio-Rad Laboratories) and transferred to PVDF membranes (Bio-Rad Laboratories). The membranes were blocked with EveryBlot Blocking Buffer (Bio-Rad Laboratories) for 5 min and then treated overnight using primary antibodies at 4 °C. All primary antibodies were diluted in the EveryBlot Blocking Buffer. Antibodies against GM130, phospho-p65 (Ser536), JAK1, phospho-STAT6, STAT6, phospho-STAT1, STAT1, phospho-STAT5 (Cell Signaling Technology, Leiden, The Netherlands), calnexin, IL-1β, p65, IL-13Rα1, TSLP, β-actin, GAPDH (Abcam, Cambridge, UK), TSG101, CD81, IL-4Rα, phospho-JAK1, CD23, FcεRI, (Invitrogen), IL-31Rα (LSBio, Seattle, WA, USA), OSMRβ, and STAT5 (R&D systems, Minneapolis, MN, USA) were used as the primary antibodies. Western blotting for all target proteins, except CD81, was performed under reducing conditions. The membranes were washed for 10 min five times and then treated with the secondary antibodies for 1 h. Anti-rabbit IgG and anti-mouse IgG (Abcam) antibodies were used as the secondary antibodies. After the membranes were washed for 10 min and repeated five times, the target proteins were detected using the ECL Select™ Western Blotting Detection Reagent (GE Healthcare, Little Chalfont, UK) and analyzed using the ChemiDoc Imaging System (Bio-Rad Laboratories).

### Histology

Approximately equal regions of individual NC/Nga mouse dorsal skin tissues were trimmed based on the sagittal axis. Skin tissues were fixed in 10% formalin for 24 h. After paraffin embedding using Shandon Citadel 2000 (Thermo Fisher Scientific) and Shandon Histostar (Thermo Fisher Scientific), 3–4 μm serial sections were made by each paraffin block using RM2255 (Leica Biosystems, Nussloch, Germany). The skin tissues were stained with hematoxylin and eosin for general histopathology, and toluidine blue stain for mast cells. Histological data of skin tissues were observed using Model Eclipse 80*i* (Nikon, Tokyo, Japan) equipped with the ProgRes™ C5 camera (Jenoptik Optical Systems GmbH, Jena, Germany) and *i*Solution FL ver 9.1 image analyzer (IMT *i*-solution Inc., Bernaby, BC, Canada).

### Cell viability assay

In 96-well plates, primary human dermal fibroblasts (5 × 10^3^ cells/mL) and HaCaT keratinocytes (3 × 10^3^ cells/mL) were seeded. After 16 h, both cells were cultivated for a further 24 h with IFN-γ-iMSC-EVs in serum-free DMEM at 37 °C and 5% CO_2_. The effects of IFN-γ-iMSC-EVs on the cell viability were assessed using the Cell Counting Kit-8 (Enzo life sciences, Farmingdale, NY, USA) according to the manufacturer’s instruction. A multiplate reader (Thermo Fisher Scientific) was used to measure the absorbance (OD value) at 450 nm.

### Statistical analyses

Statistical analyses were performed using SPSS (version 18.0 for IBM, Chicago, IL, USA). For comparisons involving three or more groups, one-way analysis of variance was performed followed by Tukey’s post hoc test. For comparisons involving only two groups, the paired Student’s t-test was used. Data are expressed as means ± standard error (SE), and values with *p* < 0.05 were considered statistically significant.

## Supplementary Information

**Additional file 1: Fig. S1.** No alteration of cell viability by IFN-γ-iMSC-EVs. Human dermal fibroblasts (left panel) and keratinocytes (right panel) were used to test the effects of cell viability on IFN-γ-iMSC-EVs. **Fig. S2.** Survival rate (A) and body weight (B) were assessed during 4 weeks following IFN-γ-iMSC-EVs treatment. **Table S7.** Sequences of primers used for real-time qPCR analysis.

**Additional file 2: Table S1.** GSEA with IFNγ-induced DEGs with the Hallmark collection. **Table S2.** GSEA with IFNγ-induced DEGs with the immunological signature collection. **Table S3.** GSEA with IFNγ-induced DEPs in EVs with the Hallmark collection. **Table S4.** GSEA with IFNg-induced DEPs in EVs with the immunological signature collection. **Table S5.** Local clustering coefficient gene sets of immune system diseases and IFNγ-induced EV proteins (D: disease, E: EV proteins).

## Data Availability

All data generated or analyzed during this study are included in this published article.

## Abbreviations

AD: Atopic dermatitis
APC: Allophycocyanin
CD: Cluster of differentiation
CerS: Ceramide synthase
CPM: Counts per million
DEG: Differentially expressed gene
DEP: Differentially expressed protein
DiR: 1,1′-Dioctadecyl-3,3,3′,3′-tetramethylindotricarbocyanine iodide
DNCB: 2, 4-Dinitrochlorobenzene
EV: Extracellular vesicle
FASN: Fatty acid synthase
FcεRI: Fc epsilon receptor I
GM130: Golgi matrix protein 130
GSEA: Gene set enrichment analysis
HMGCR: 3-Hydroxy-3-methylglutaryl-CoA reductase
IDO1: Indoleamine 2,3-dioxygenase 1
IFN-γ: Interferon-gamma
IL-4Rα: Interleukin 4 receptor alpha subunit
IL-13Rα1: Interleukin 13 alpha 1 subunit
IL-31Rα: Interleukin-31 receptor alpha subunit
iMSC: Induced mesenchymal stem cells
nm: Nanometer
JAK1: Janus kinase 1
KRT: Keratin
MS: Multiple sclerosis
NTA: Nanoparticle tracking analysis
OSMRβ: Oncostatin M receptor beta subunit
PBS: Phosphate buffered saline
RU: Relative unit
SCID: Severe combined immunodeficiency
SE: Standard error
SJS: Steven–Johnson syndrome
SPT: Serine palmitoyl transferase
STAT: Signal transducer and activator of transcription
TEM: Transmission electron microscopy
TSG101: Tumor susceptibility gene 101
TSLP: Thymic stromal lymphopoietin
TEWL: Transepidermal water loss
