# Supplementary material for: Extracellular vesicles from IFN-γ-primed mesenchymal stem cells repress atopic dermatitis in mice
Source: J Nanobiotechnology. 2022 Dec 10;20:526. doi: 10.1186/s12951-022-01728-8 (PMC9741801; doi:10.1186/s12951-022-01728-8)
Supplement: Supplementary file 1 — Additional file 1: Fig. S1. No alteration of cell viability by IFN-γ-iMSC-EVs. Human dermal fibroblasts (left panel) and keratinocytes (right panel) were used to test the effects of cell viability on IFN-γ-iMSC-EVs. Fig. S2. Survival rate (A) and body weight (B) were assessed during 4 weeks following IFN-γ-iMSC-EVs treatment. Table S7. Sequences of primers used for real-time qPCR analysis. [file 12951_2022_1728_MOESM1_ESM.pdf]

Additional file 1

Extracellular vesicles from IFN-γ-primed mesenchymal stem cells repress atopic dermatitis in mice

Jimin Kim[1], Seul Ki Lee[1], Minyoung Jung[1], Seon-Yeong Jeong[1], Haedeun You[1], Ji-Yeon Won[1], Sang-Deok Han[1], Hye Jin Cho[1], Somi Park[1], Joonghoon Park[2,3], Tae Min Kim[2,3*], Soo Kim[1*]

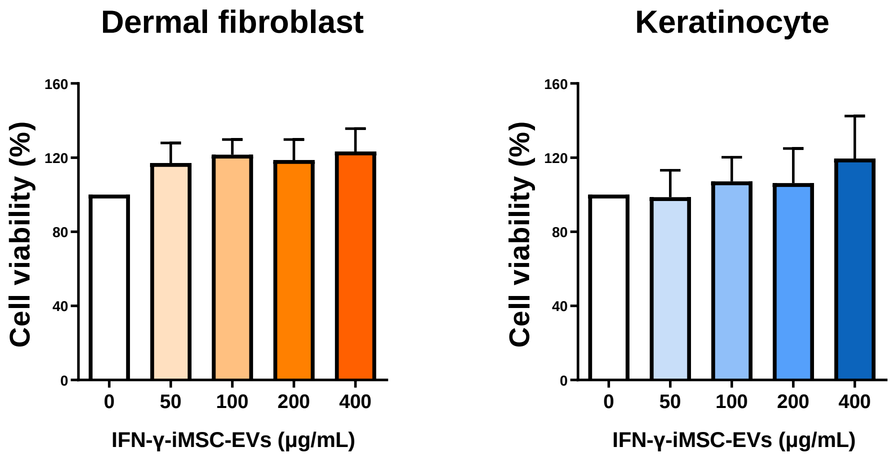

Fig. S1 No alteration of cell viability by IFN-γ-iMSC-EVs. Human dermal fibroblasts (left panel) and keratinocytes (right panel) were used to test the effects of cell viability on IFN-γ-iMSC-EVs.

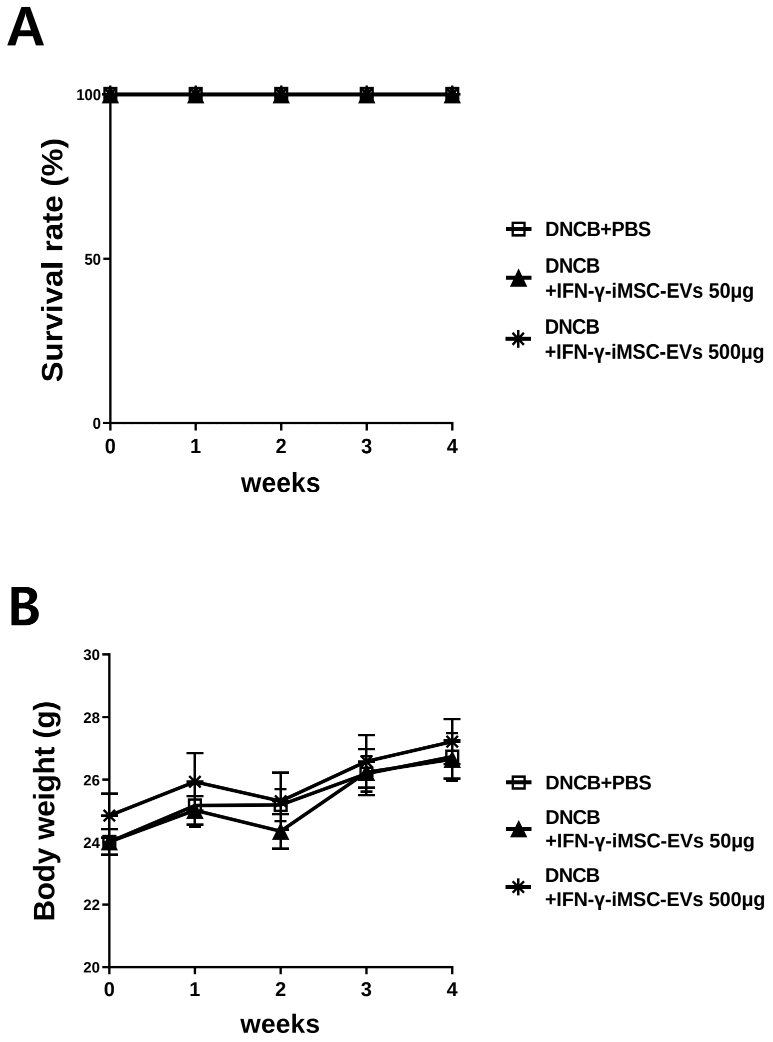

Fig. S2 Survival rate (A) and body weight (B) were assessed during 4 weeks following IFN-γ-iMSC-EVs treatment.

Table S7. Sequences of primers used for real-time qPCR analysis.

Gene	Forward (5'–3')	Reverse (5'–3')
GAPDH	ACATCGCTCAGACACCATG	TGTAGTTGAGGTCAATGAAG
IDO1	GCCCTTCAAGTGTTTCACCAA	GCCTTTCCAGCCAGACAAATAT
